# Sonographic Gallbladder Abnormality Is Associated with Intravenous Immunoglobulin Resistance in Kawasaki Disease

**DOI:** 10.1100/2012/485758

**Published:** 2012-06-18

**Authors:** Chih-Jen Chen, Fu-Chen Huang, Mao-Meng Tiao, Ying-Hsien Huang, Li-Yan Lin, Hong-Ren Yu, Kuender D. Yang, Yi-Chuan Huang, Chih-Cheng Chen, Wei-Chiao Chang, Ho-Chang Kuo

**Affiliations:** ^1^Department of Pediatrics, Kaohsiung Chang Gung Memorial Hospital, Chang Gung University College of Medicine, Kaohsiung, Taiwan; ^2^Department of Medical Research and Pediatrics, Show Chwan Memorial Hospital in Chang Bing, Changhua, Taiwan; ^3^School of Pharmacy, College of Pharmacy, Taipei Medical University, Taipei, Taiwan; ^4^Cancer Center, Kaohsiung Medical University Hospital, Kaohsiung, Taiwan; ^5^Department of Medical Genetics, Kaohsiung Medical University, Kaohsiung, Taiwan

## Abstract

*Objective*. Kawasaki disease (KD) is an acute systematic vasculitis in children which causes coronary arterial lesions and hydrops of gallbladder. Our objective is to correlate the clinical significance and influence on disease outcome of patients with gallbladder abnormalities in Kawasaki dissease. *Methods*. Children who met KD diagnosis criteria and were admitted for IVIG treatment were retrospectively enrolled for analysis. Patients with abdominal sonography were divided into 2 groups based on the absence (Group A, *N* = 61) or presence (Group B, *N* = 16) of gallbladder abnormalities (GBA), defined as hydrops or acalculous cholecystitis. Between the two groups, clinical features, demographic data (including admission days, coronary artery lesions, IVIG resistance), and laboratory data before/after IVIG treatment were collected for analysis. *Results*. The presence of sonographic gallbladder abnormalities is correlated with higher levels of serum CRP, GPT, and neutrophils. It also points to an increased number of IVIG resistance rates in group B. There was no significant statistical difference among clinical features, age, gender, admission days, or coronary artery lesions between the two groups. *Conclusion*. Sonographic gallbladder abnormalities are associated with higher CRP, GPT, neutrophil and IVIG resistance in KD. It can be used as a predictor of IVIG resistance in patients with KD.

## 1. Introduction

Kawasaki disease (KD) is an acute multisystemic vasculitis syndrome of unknown etiology first described by Tomisaku Kawasaki et al. more than 40 years ago in Japan [1]. Since then, it has surpassed rheumatic fever to become the leading cause of acquired heart disease in children living in developed countries [2–4]. Typical diagnostic criteria for KD include an illness unexplained by another disease, fever (at least five days), and four of the five following conditions: bilateral nonpurulent conjunctival injection, oral mucosal changes (erythema or dryness or fissuring of the lips, strawberry tongue, and erythema of the oropharynx), peripheral extremity changes (edema or erythema of palms or soles, desquamation of tips of fingers and toes), rash (polymorphic and nonvesicular, commonly truncal), and cervical lymphadenopathy (over 1.5 cm) [1, 5, 6]. However, a wide range of other atypical clinical features which are not included in the diagnostic criteria such as uveitis, aseptic meningitis, urethritis, arthralgia, arthritis, abdominal pain, liver function impairment, heart failure, and gallbladder hydrops have been widely recognized as well [7–9]. The most severe complication of KD is coronary artery lesion (CAL) [9] (such as coronary artery aneurysm, coronary artery fistula [10]…) and is the leading cause of acquired heart disease of children [3, 4, 10]. High-dose intravenous immunoglobulin (IVIG) therapy (2 g/kg) with high-dose aspirin (80~100 mg/kg/day) is the standard treatment for KD, and it decreased the incidence of the coronary artery aneurysm from 20% to 3–5% [8, 11–13].

Previous research has pointed out the relatively self-limited nature and laboratory features of acute hydrops of the gallbladder (AHGB) in KD [14, 15]. Reported with less frequency but of equal importance is the clinical finding of acute acalculous cholecystitis (ACC) in KD patients [16–19]. Whether the presence of sonographic gallbladder abnormalities (AHGB and ACC) that are seen in KD patients has an influence on disease outcome is an area of interest that has rarely been explored. Thus, this study was conducted to investigate the correlation between the clinical significance of gallbladder abnormalities and its influence on disease outcome of Kawasaki disease.

## 2. Methods

### 2.1. Study Design and Participants

From 2005 to 2007, the medical charts of children who fulfilled the criteria for KD [6] and were treated with IVIG at Kaohsiung Chang Gung Memorial Hospital were analyzed retrospectively. This study was approved by the Institutional Review Board of Chang Gung Memorial Hospital. Patients whose symptoms did not fit the KD criteria, did not perform abdominal ultrasonography, or did not have complete clinical or laboratory data were excluded. Patients were initially treated with a single dose of IVIG (2 g/kg) during a 12-h our period. Aspirin was also given at an anti-inflammatory dose until the fever subsided and then switched to 3–5 mg/kg/day as a single daily dose. Low-dose aspirin was continued until all signs of inflammation subsided and the CAL (coronary arterial lesion) regressed as detected by two-dimensional (2D) echocardiography as our previous reports [22, 23]. CAL formation was defined as the internal diameter of the coronary artery measuring at least 3 mm or the internal diameter of a segment at least 1.5 times that of an adjacent segment, as observed in the echocardiogram [11, 24].

Demographic data and the principal clinical features of KD [6] that occur in the acute stage were recorded. Laboratory data, including complete blood count (CBC), differential count (DC), hemoglobin (Hb), platelet (Plt), aspartate aminotransferase (AST), alanine aminotransferase (ALT), and C-reactive protein (CRP), were included for analysis. Laboratory data was obtained before IVIG administration (before IVIG) and 3 days after completing the initial IVIG treatment (after IVIG). IVIG resistance is defined as a return of fever associated with one or more of the initial symptoms that led to the diagnosis of KD within 2–7 days after the initial IVIG treatment and included patients who needed a second dose of IVIG (1-2 g/kg) due to initial IVIG treatment failure [8, 25].

### 2.2. Sonographic Examination and Diagnostic Criteria for Gallbladder Abnormality

Patients with abdominal sonography were divided into 2 groups based on the absence (group A) or presence (group B) of gallbladder abnormalities (hydrops or acalculous cholecystitis). Hydrops is defined as an enlargement (longitudinal and horizontal diameter greater than that for age-matched average values) without changes in normal anatomical characteristics (i.e., without sludge, stone, increased wall thickness, or pericholecystic fluid) of the gallbladder [26–28]. Whereby ultrasonographic criteria for acute acalculous cholecystitis consisted of two out of the following four characteristics: (1) distention of the gallbladder, (2) gallbladder wall thickness greater than 3.5 mm, (3) presence of sludge, and (4) pericholecystic fluid collection [29–31]. Between the two groups, demographic data (including admission days, CAL, and initial IVIG resistance), clinical features, and laboratory data before/after IVIG treatment were collected for analysis.

### 2.3. Statistical Analysis

Comparison of continuous data (mean ± standard deviation) was calculated by Student's *t*-tests. The median values of each parameter were used as cutoff values. Univariate analysis with chi-square test and multivariate analysis with logistic regression were used to assess the parameters between the two groups. A *P* value <0.05 was accepted as statistically significant. All statistical tests were performed using spss 13.0 for Windows (SPSS Inc., Chicago, IL, USA).

## 3. Results

A total of 93 children were diagnosed with KD during the study period. Out of these, 77 [53 boys (69%) and 24 girls (31%)] had performed abdominal sonography and were enrolled for further analysis. Sixteen (21%) of the 77 children had abnormal gallbladder findings, 11 (69%) were acute hydrops of gallbladder (AHGB), and 5 (31%) were acute acalculous cholecystitis (ACC). 75% (12/16) of those with positive findings were boys. None had sonographic evidence of pancreatic pathology or intra/extrahepatic biliary tree dilatations. Demographic characteristics, clinical manifestations, and laboratory findings before and after IVIG treatments are shown in Table 1 through 4. There were no significant differences between the two groups in terms of age, total admission days, coronary artery lesion involvement, or clinical features (Tables 1 and 2). We found that the presence of sonographic gallbladder abnormalities (GBAs) is correlated with initial IVIG resistance (7/61 versus 6/16, *P* = 0.023), higher levels of serum CRP (94.6 ± 77.4 versus 143.9 ± 78.2 mg/L, *P* = 0.027), GPT (81.2 ± 100.1 versus 150.9 ± 133.5 U/L, *P* = 0.028), neutrophils (61.9 ± 15.2 versus 76.8 ± 10.2%, *P* < 0.001), and lower levels of lymphocytes (27.4 ± 14.4 versus 14.8 ± 9.10%, *P* < 0.001) (Tables 3 and 4). Lymphocyte and platelet counts tended to increase in both groups after IVIG treatment, albeit without statistical significance (45.8 ± 18.8 versus 33.2 ± 23.2, *P* = 0.26) for lymphocyte, but with significance for platelet count (47.75 ± 16.62 versus 35.41 ± 12.61 × 10^4^/mm^3^, *P* = 0.007).

A multivariate analysis using significant parameters namely gallbladder abnormality, pre IVIG neutrophil, lymphocyte, CRP, GPT and post IVIG platelet, neutrophil, CRP, GPT was performed to see it's correlation with IVIG resistance. Only 2 independent variables, GBA and post IVIG platelet count, *P* = 0.018 and *P* = 0.013 (both with confidence interval of 95%, range 1.382 to 29.630 for GBA and 1.016 to 1.14 for platelet count respectively) were identified to be significantly associated with IVIG resistance. 

Lastly, the group with GBA was subdivided into two groups, acute hydrops of gallbladder (*N* = 11) group and acalculous cholecystitis (*N* = 5) group. There were no significant differences among patient characteristics, admission days, CAL involvement, IVIG resistance, clinical features, or laboratory findings between these two groups (Table 5 and other data not shown).

## 4. Discussion

The association between KD and hydrops of the gallbladder has been reported as early as the late 1970s and early 1980s in the form of isolated case reports [15, 21, 26, 32–34]. Due to the advent of the gray-scale ultrasound, more and more cases of hydrops of the gallbladder were being diagnosed and recognized as an occasional clinical entity of KD with an incidence rate of 5–14% [14, 15]. In our study, 21% (16/77) of KD patients who performed abdominal sonography had hydrops or acalculous cholecystitis, with 31% (5/16) being that of acute acalculous cholecystitis. Only a few handful of reports have correlated the finding of acalculous cholecystitis with KD [16–19], nevertheless, as is evident in our study and those presented in the literature, it is still a relevant finding.

One of the most dreaded complication of KD is the involvement of the coronary arteries, with sequelae such as myocardial infarction, coronary artery fistula formation, coronary artery dilatation, and coronary artery aneurysms [6, 10]. Fortunately, a highly effective treatment with IVIG and aspirin could lower the incidence of coronary aneurysm formation from 20–25% in those who are untreated to just 3–5% in treated cases [12, 35]. Though the treatment with IVIG is effective, 10–20% (with some reporting up to 38% [36]) of cases will be resistant to initial IVIG therapy, and these are at increased risk for coronary artery lesions and morbidity/mortality [12, 20, 25, 37, 38].

In our study, there were no significant differences between the gallbladder abnormality and nongallbladder abnormality groups in terms of age, sex, coronary artery involvement, or total admission days. However, the group of GBA is associated with a higher level of neutrophils, GPT, and CRP. This finding is comparable with those reported previously in the literature [26, 33, 34]. Of special interest and novel finding is the association between the presence of sonographic GBA with that of initial IVIG resistance. Previous research on the risks of IVIG resistance in KD [39, 40] also reported increased levels of neutrophils, CRP, GPT, and lower platelet counts, the same laboratory parameters that were found in our study. This makes perfect sense since this group is associated with increased risk of IVIG resistance.

Although the pathophysiology behind these gallbladder abnormalities may be different, they may share a common root in KD. Currently, the etiology behind both AHGB and ACC in KD is unknown, however; common to both is the proposed mechanism of (1) adenopathy around the cystic duct causing obstruction, (2) vasculitis or perivasculitis of the gallbladder wall, and (3) inflammatory infiltrates with polymorphs, lymphocytes, and eosinophils [15, 30, 34, 41]. Previously unaware of the relatively benign and self-limited nature of gallbladder hydrops and acute acalculous cholecystitis in KD, these changes were observed in the pathological specimens of gallbladders resected from KD patients in the late 1980s [15, 28].

## 5. Conclusion

To the best of our knowledge, the association between the presence of sonographic GBA and that of initial IVIG resistance in KD has never been reported. In addition to the previously known laboratory values that have been associated with IVIG resistance [42], we provide an additional “Visual” parameter that can be used as a supplement to the risk-scoring system [43]. This finding can alert the physician, providing care for a group of KD patients which are at an increased risk for developing coronary artery lesions, to plan ahead and make the necessary adjustments to treatment.

## Figures and Tables

**Table 1:** Characteristic of gallbladder abnormalities (GBAs) and non-GBA in Kawasaki disease patients.

	Group A (non-GBA, *N* = 61)	Group B (GBA, *N* = 16)	*P* value
Age (m/o)	20.4 ± 18.9	28.7 ± 16.8	0.116
Male gender (%)	41 (67%)	12 (75%)	0.549
Admission days	7.75 ± 3.91	6.83 ± 3.23	0.295
IVIG resistance (%)	7 (11)	6 (38)	0.023*
CAL (%)	20 (33)	6 (38)	0.771

CAL: coronary artery lesion; IVIG: intravenous immunoglobulin. *P < 0.05.

**Table 2:** Principal clinical features in the acute stage of Kawasaki disease between gallbladder abnormalities (GBAs) and non-GBA groups.

	Group A (non-GBA)	Group B (GBA)	*P* value
Neck lymphadenopathy	26/60 (43%)	7/14 (50%)	NS
Fissured lips	53/60 (88%)	12/14 (86%)	NS
Strawberry tongue	47/61 (77%)	11/14 (79%)	NS
Conjunctivitis	55/60 (92%)	12/14 (86%)	NS
Skin rash	51/58 (88%)	15/15 (100%)	NS
BCG erythema change	25/58 (43%)	6/13 (46%)	NS
Induration of extremities	39/56 (70%)	3/12 (25%)	NS

NS: nonsignificance.

**Table 3:** Comparison of lab data between gallbladder abnormality (GBA) and non-GBA in Kawasaki disease before IVIG therapy.

	Group A (non-GBA, *N* = 61)	Group B (GBA, B = 16)	*P* value
WBC cell (/mm^3^)	13854 ± 5005	12519 ± 4465	0.335
Hemoglobin (g/dL)	11.0 ± 1.33	11.0 ± 1.23	0.948
Platelet (×10^4^/mm^3^)	35.4 ± 13.0	30.4 ± 9.03	0.156
Neutrophil (%)	61.9 ± 15.2	76.8 ± 10.2	0.001*
Lymphocyte (%)	27.4 ± 14.4	14.8 ± 9.10	0.001*
Eosinophil (%)	2.62 ± 2.82	1.39 ± 2.01	0.108
Monocyte (%)	6.51 ± 3.51	5.86 ± 2.84	0.497
C-reactive protein (mg/L)	94.6 ± 77.4	143.9 ± 78.2	0.027*
GOT (U/L)	58.8 ± 72.9	78.3 ± 80.6	0.358
GPT (U/L)	81.2 ± 100	150.9 ± 133.5	0.028*

*P < 0.05.

**Table 4:** Comparison of lab data between GBA and non-GBA in Kawasaki disease after IVIG therapy.

	Group A (non-GBA, *N* = 61)	Group B (GBA, *N* = 16)	*P* value
WBC cell (/mm^3^)	9849 ± 3693	10662 ± 3923	0.441
Hemoglobin (g/dL)	10.6 ± 1.33	10.4 ± 1.02	0.564
Platelet (×10^4^/mm^3^)	47.8 ± 16.6	35.4 ± 12.6	0.007*
Neutrophil (%)	40.3 ± 18.2	53.6 ± 24.6	0.018*
Lymphocyte (%)	45.8 ± 18.8	33.2 ± 23.19	0.26
Eosinophil (%)	4.05 ± 5.08	5.02 ± 3.40	0.473
Monocyte (%)	7.40 ± 3.03	6.01 ± 3.21	0.113
C-reactive protein (mg/L)	38.9 ± 36.8	85.7 ± 71.1	0.032*
GOT (U/L)	30.0 ± 7.78	39.7 ± 17.7	0.084
GPT (U/L)	35.7 ± 19.0	68.7 ± 40.5	0.016*

*P < 0.05.

**Table 5:** Characteristic of AHGB versus ACC in Kawasaki disease patients.

	AHGB (*N* = 11)	ACC (*N* = 5)	*P* value
Age (m/o)	25.41 ± 15.03	35.80 ± 19.94	0.265
Male gender (%)	9 (82%)	3 (60%)	1.000
Admission days	7.22 ± 3.56	8.00 ± 2.94	0.711
IVIG resistance	4	2	0.604
CAL	3	3	0.299

AHGB = acute hydrops of gallbladder; ACC = acute acalculous cholecystitis; CAL: coronary artery lesion; IVIG: intravenous immunoglobulin.
